# Proteomic Analyses of Human Cytomegalovirus Strain AD169 Derivatives Reveal Highly Conserved Patterns of Viral and Cellular Proteins in Infected Fibroblasts

**DOI:** 10.3390/v6010172

**Published:** 2014-01-07

**Authors:** Sabine Reyda, Nicole Büscher, Stefan Tenzer, Bodo Plachter

**Affiliations:** 1 Institute for Virology, University Medical Center of the Johannes Gutenberg-University Mainz, Obere Zahlbacher Str. 67, D-55131 Mainz, Germany; E-Mails: reyda@uni-mainz.de (S.R.); bueschni@uni-manz.de (N.B.); 2 Institute for Immunology, University Medical Center of the Johannes Gutenberg-University Mainz, Langenbeckstr. 1, D-55131 Mainz, Germany; E-Mail: tenzer@uni-mainz.de; 3 Research Center Immunology, University Medical Center of the Johannes Gutenberg-University Mainz, Langenbeckstr. 1, D-55131 Mainz, Germany

**Keywords:** human cytomegalovirus, proteomics, mass spectrometry, virions, expression pattern

## Abstract

Human cytomegalovirus (HCMV) particle morphogenesis in infected cells is an orchestrated process that eventually results in the release of enveloped virions. Proteomic analysis has been employed to reveal the complexity in the protein composition of these extracellular particles. Only limited information is however available regarding the proteome of infected cells preceding the release of HCMV virions. We used quantitative mass spectrometry to address the pattern of viral and cellular proteins in cells, infected with derivatives of the AD169 laboratory strain. Our analyses revealed a remarkable conservation in the patterns of viral and of abundant cellular proteins in cells, infected for 2 hours, 2 days, or 4 days. Most viral proteins increased in abundance as the infection progressed over time. Of the proteins that were reliably detectable by mass spectrometry, only IE1 (pUL123), pTRS1, and pIRS1 were downregulated at 4 days after infection. In addition, little variation of viral proteins in the virions of the different viruses was detectable, independent of the expression of the major tegument protein pp65. Taken together these data suggest that there is little variation in the expression program of viral and cellular proteins in cells infected with related HCMVs, resulting in a conserved pattern of viral proteins ultimately associated with extracellular virions.

## 1. Introduction

The human cytomegalovirus (HCMV) is a pathogen of substantial clinical relevance that may lead to severe disease and sequelae after prenatal infection and life threating conditions in immunosuppressed individuals. Considerable interest thus focuses on the development of therapeutic strategies against HCMV infection and disease. One target area for the development of antiviral compounds is particle formation and particle release. 

The virions of HCMV are made up by an inner capsid structure, containing the linear double-stranded DNA genome of roughly 235 kbp, an attached matrix of viral and cellular proteins, assembled as a tegument layer, and an outer envelope [1]. The 100 nm icosahedral capsid is composed of the major capsid protein (MCP, pUL86), the minor capsid protein (mCP, pUL85), the mCP-binding protein (mCP-BP, pUL46), the smallest capsid protein (SCP; pUL48.5), and the portal protein (pUL104) [2]. Assembly of the capsids proceeds in the nucleus of infected cells, originating from scaffold-containing procapsids. The viral DNA is encapsidated and selected tegument proteins may become associated with the capsids already at this stage [1,2]. Capsids exit the nucleus by subsequent envelopment and de-envelopment at the nuclear membrane. A key determinant of that process is the nuclear egress complex (NEC) at the inner nuclear membrane that consists of the viral proteins pUL50 and pUL53 [3,4,5,6]. Nuclear capsid egress appears to be regulated by both cellular and viral kinases [3,7,8]. Following their release into the cytoplasm, the capsids are directed to perinuclear inclusions termed assembly compartments (AC) [9]. The AC consists of cylindrical structures that are assembled from the trans-Golgi network, the endoplasmic reticulum (ER), the ER-Golgi intermediate compartment (ERGIC) and from endosomal compartments, and contains proteins characteristic for endosomal sorting and transport (ESCRT) as well as Rab GTPases [1,10,11,12]. Tegument attachment to the capsids occurs predominantly at the AC and precedes envelopment. Both virions and subviral dense bodies are enveloped at AC and are subsequently found in cytoplasmic vesicles that are then transported to the cell surface by an exocytotic pathway [6,13]. This transport is influenced by the product of the viral UL103 open reading frame (ORF) [14]. Infectious virions and dense bodies are ultimately released at the cell surface. 

Early analyses used color staining or radioactive labeling in combination with SDS-PAGE to display the protein pattern of purified HCMV virions [15,16,17,18]. Subsequent microsequencing analyses identified a subset of viral and cellular protein components of HCMV virions [19]. Further reports also identified cell derived proteins to be included in HCMV virions, although some of these results have later been challenged as being a result of contamination rather than of packaging [20,21,22,23,24]. A previous study, using mass spectrometry, revealed the high level of complexity of the HCMV virion [25,26]. Seventy-one viral proteins were identified to be integral constituents of the particle. In addition, over 70 cell-derived proteins were found to be associated with purified particles. Proteomic analyses of the murine CMV virion displayed a similarly complex picture [27].

Mass spectrometry has provided a detailed insight into the protein composition of HCMV virions. There is, however, only limited information available about the proteomic composition of permissively infected fibroblasts preceding the release of particles. Furthermore, analyses of the protein composition of HCMV virions by mass spectrometry were focused on one single AD169-derivative. Up to this point, it remained unclear how culturing of related HCMVs in different laboratories would influence the proteomes of infected cells and virions. To begin to address this issue, we focused on five HCMVs that were all descendants, as bacterial artificial chromosome (BAC) clones, of the AD169 laboratory strain of HCMV. The viruses used for analysis were RV-HB5 [28], RV-HB15 (AD169-RV) [29]) and RV-BADwt [30], and the pp65 deletion mutants RV-Hd65 [31], RV-KB14 [32]. The RV-HB5 is the first BAC-derived HCMV strain, originally cloned by Borst and colleagues by inserting a BAC-vector into the US2-US6 gene region of the AD169 strain [28]. The RV-HB15 is a modified version of RV‑HB5, where the US2-US6 deletion was repaired and the BAC vector was removed by cre-lox recombination at virus reconstitution, leaving one single loxP site behind in the genome. [29]. The RV‑BADwt is a full length AD169 strain that was cloned by inserting a self-excisable BAC-vector between the ORFs US28 and US29, again leaving one single loxP site behind, following the US28 ORF [30]. The pp65neg strain RV-Hd65 was derived by replacing the UL83 (pp65) ORF from AD169‑BAC by a neomycin resistance cassette, leaving behind 151 5'-terminal base pairs of the pp65 ORF [31]. The AD169-BAC is the clone used to reconstitute RV-HB15. The neomycin resistance cassette was removed using FLP-recombinase mediated excision in *E. coli.* The BAC vector was removed by cre‑lox recombination at virus reconstitution. The pp65neg strain RV-KB14 was generated by inserting a tetracycline resistance cassette into the UL83 (pp65) ORF of pAD/cre (the BAC clone used for reconstitution of RV-BADwt), thereby deleting all of the pp65-coding region except for 152 5' base pairs of the ORF [32]. All viruses were characterized before with respect to their genomic structure and biologic properties. 

The data analyses presented here show a remarkable conservation of the overall levels of viral and cellular proteins in fibroblasts, infected with RV-HB15 or RV-BADwt. Most viral proteins increased in their steady-state levels in infected cells up to 4 days after infection (dpi). Only IE1 (pUL123), IRS1 and TRS1 appeared to be downregulated at 4 dpi. No gross alteration of the viral protein content of the virions of pp65pos- or pp65neg-viruses were seen. 

## 2. Results and Discussion

### 2.1. Conserved Pattern of Viral Protein Expression in Infected Cells

To address the steady-state levels of viral proteins in HFF, cells were infected at an m.o.i. of 1 with RV-HB15 and RV-BAD, respectively. Cells were collected and processed after 2 hpi, 2 dpi, or 4 dpi, respectively, and analyzed by mass spectrometry.

**Figure 1:**
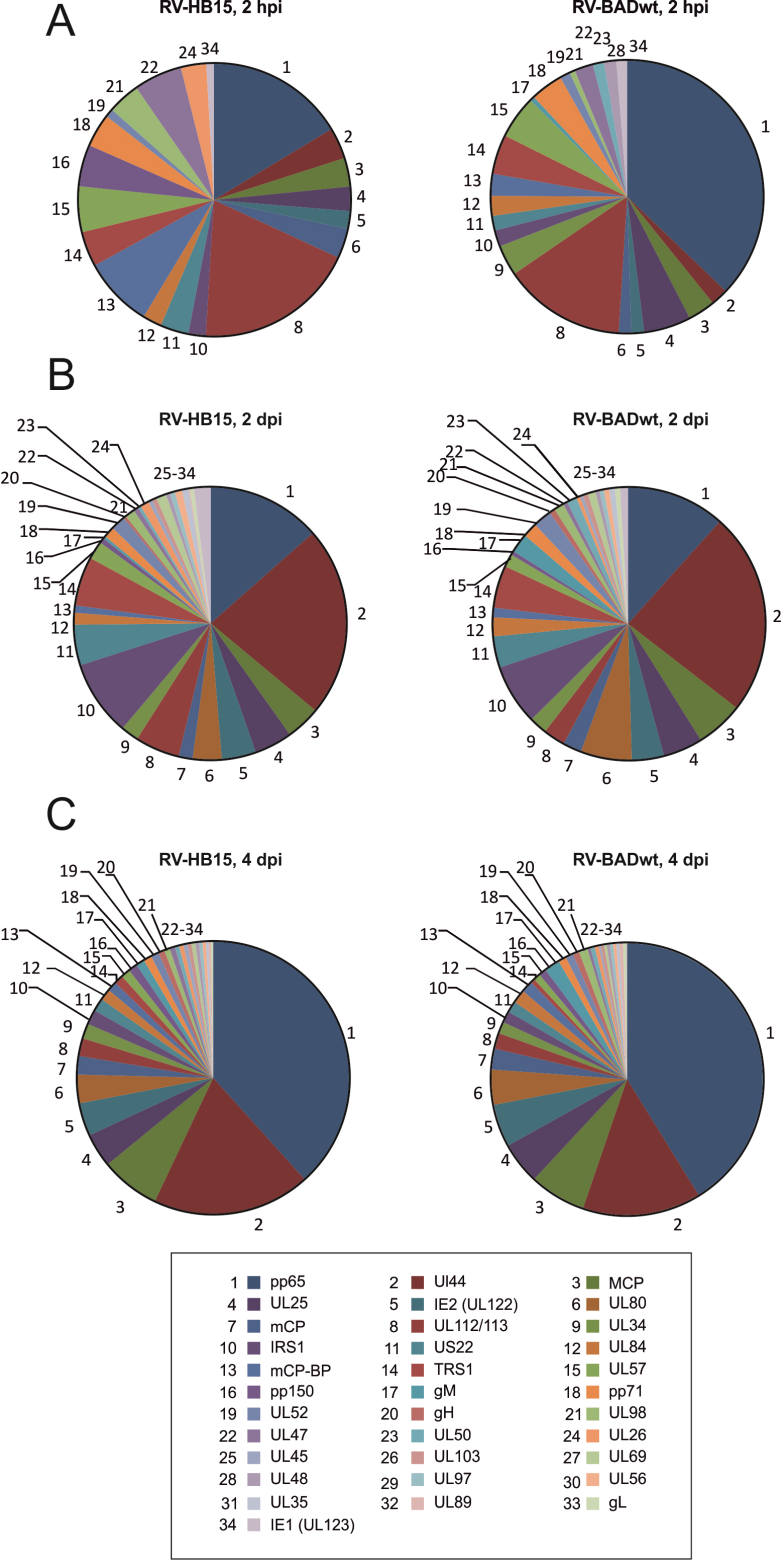
Proteomic analysis of viral proteins expressed in infected HFF. Fibroblasts were infected with RV-HB15 or RV-BADwt. At 2 hpi, 2 dpi, and 4 dpi, the cells were collected and analyzed, using nanoUPLC mass spectrometry. The relative abundance of individual proteins is shown in the pie charts. TOP3-Intensity was calculated as the average intensity of the three best ionizing peptides and is proportional to the molar amount of the respective protein in the sample. Proteins were sorted in decreasing abundance according to the values determined for four-day RV-HB15 infected fibroblasts and numbered accordingly, as indicated in the box. (**A**) samples obtained for analysis at 2 hpi; (**B**) samples obtained for analysis at 2 dpi; (**C**) samples obtained for analysis at 4 dpi.

**Figure 2:**
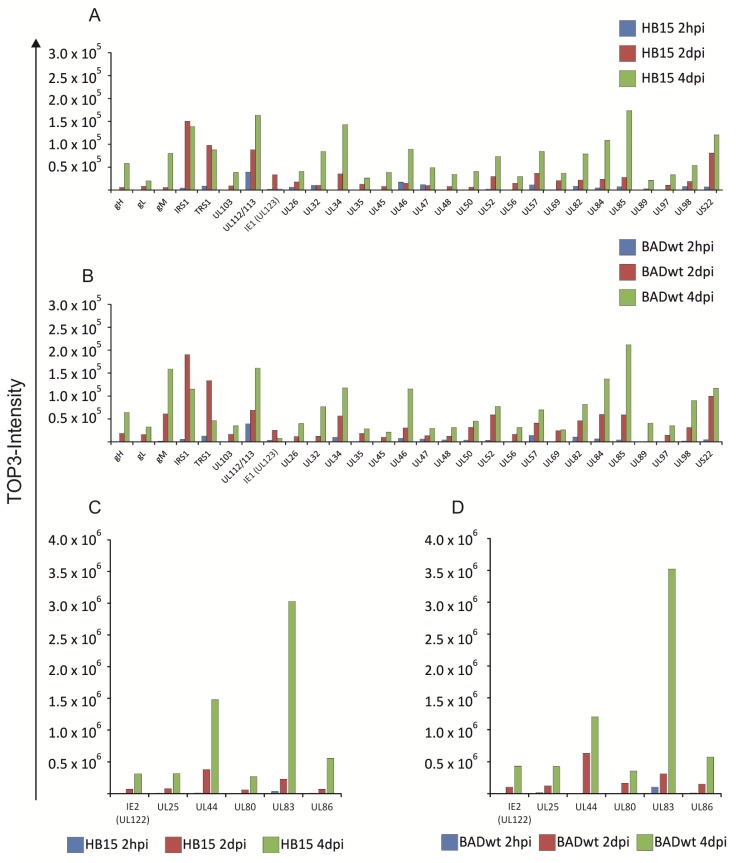
Time course of viral protein levels in infected HFF. The data shown in the pie charts in Figure 1 are displayed in bar chart format to show the course for each individual protein. (**A**) and (**C**), viral proteins in RV-HB15 infected cells. (**B**) and (**D**), viral proteins in RV‑BADwt infected cells. Note that the scales are different in (**A**) and (**B**) *versus* (**C**) and (**D**). TOP3-intensity was calculated as the average intensity of three best ionizing peptides and is proportional to the molar amount of the respective protein in the sample.

The patterns of viral proteins that were detectable at 2 hpi were divergent between RV-HB15 and RV-BADwt (Figure 1A). This, however, was not surprising, considering the low amounts of viral proteins relative to the prevalence of cellular proteins at this early time after infection (data not shown). The precision of the measurement of viral proteins against this high background was limited, thus compromising a direct comparison in this case. It was yet remarkable that viral proteins could be detected at this early time after infection, underscoring the sensitivity of the proteomic approach. In contrast to these results, the patterns of viral proteins at 2 dpi were comparable between RV-HB15 and RV-BADwt (Figure 1B). This indicated that the protein expression of these two viruses was highly conserved over the passaging on human fibroblasts. This was corroborated by the patterns observed at 4 dpi (Figure 1C). As expected, the pattern changed from 2 to 4 dpi for both viruses. At 2 hpi and 2 dpi, regulatory proteins, like IRS1, or proteins for DNA replication, like UL44 were most prominent (Figure 2 and Table 1). Note that, at this time point, pUL44 was the most abundant protein in infected cells. The levels of the regulatory proteins IE1 (UL123), TRS1 and IRS1 already peaked at 2 dpi. These three were the only viral proteins that appeared to be downregulated, resulting in a decreased abundance at 4 dpi (Figure 2). At the later time point, structural proteins more prominently shaped the pattern of expression in HCMV infected cells, pp65 being by far the most abundant representative. Surprisingly, however, UL44 was also highly expressed at 4 dpi, although other proteins, involved in DNA replication did not reach that level. The reason for this abundance of UL44 in HCMV infected fibroblasts is unclear at this point.

The proteomic approach shown here provided an impressive reflection of the long known burst of viral protein expression in infected fibroblasts. Whereas viral proteins constituted below 1% of the total protein mass in infected cells at 2 hpi, this increased to over 20% at 4 dpi (data not shown). Given the long half-life of many abundant cellular proteins, these results underscore the intensity with which HCMV diverts the cellular biosynthesis to its own use. 

### 2.2. Comparable Impact of RV-HB15- and RV-BADwt-Infection on the Cellular Proteome

**Figure 3:**
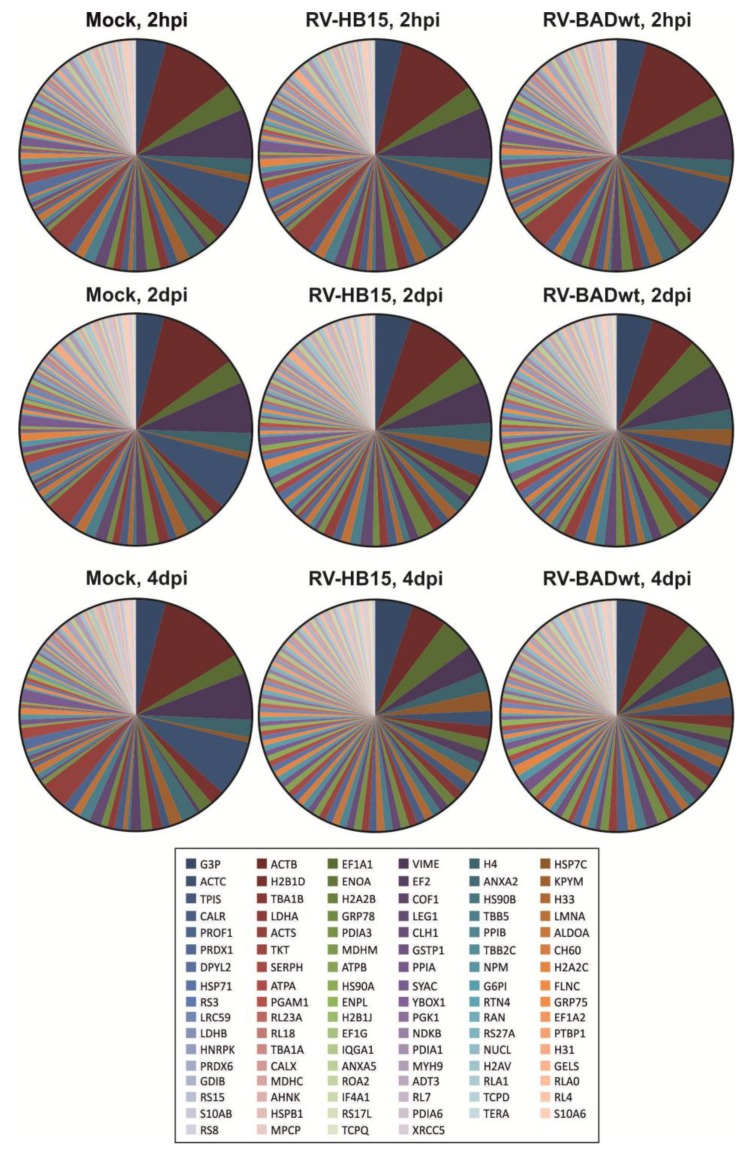
Proteomic analysis of cellular proteins in infected HFF. Fibroblasts were infected with RV-HB15 or RV-BADwt. After 2 hpi, 2 dpi or 4 dpi, the cells were collected and analyzed using nanoUPLC mass spectrometry. The relative frequency of the viral proteins in the samples is shown in the pie charts. Proteins were sorted according to frequencies found in four day RV-HB15 infected fibroblasts, displayed from left to right in the caption. The 100 most frequent proteins in RV-HB15 infected HFF are shown. A detailed representation of the data is provided in the Supplementary Table 1.

Proteomic analyses of the expression patterns of viral proteins in infected HFF displayed relatively conserved patterns between two different AD169 derived viruses. To test the influence of the two viruses on the levels of cell proteins in infected HFF, the mass spectrometry data of infected fibroblasts were analyzed with regard to cellular proteins (Figure 3 and Supplementary Table 1). Uninfected cells (mock) that were collected at different times after passage showed a proteomic pattern that displayed little change over time. This result compellingly documented the accuracy and the reproducibility of the method. The protein pattern of cells that were infected for 2 hours was identical to the pattern of mock infected HFF for both tested viruses. This was not surprising, as no gross changes were expected to occur at this early time. The pattern started to change at 2 dpi, and these changes were even more pronounced at 4 dpi. The alterations were consistent with the results of many reports detailing the dramatic impact of HCMV infection on cellular gene expression [1,33,34]. Little differences were, however, seen between RV-HB15- and RV-BADwt-infected cells at any of the tested time points. This indicates that HCMV infection with AD169-derived viruses results in a rather uniform pattern of changes in the infected cell proteome (details of the data set can be found in Supplementary Table 1). Further analyses are, however, required to investigate if the pattern of cell protein expression varies, when viruses other than AD169-derivatives are analyzed. In addition the experimental setup chosen here can only provide a pattern-analysis of the infected cell proteome. A more detailed insight into the HCMV-induced changes of particular host cell proteins will await further analyses. 

**Table 1:** Viral proteins detected by nanoUPLC mass spectrometry in infected cells.

Capsid proteins									
HCMV ORF	Synonym	Max score ^a^	Reported peptides^ b^	RV-HB15	RV-HB15	RV-HB15	RV-BADwt	RV-BADwt	RV-BADwt
				2 hpi^ c^	2 dpi ^c^	4 dpi ^c^	2 hpi ^c^	2 dpi ^c^	4 dpi^ c^
UL46	mCP-BP	7,323.97	9	17,316	14,320	89,186	7,064	30,425	115,613
UL85	mCP	11,871.54	12	7,257	27,654	173,498	4,010	58,905	211,460
UL86	MCP	29,574.99	42	6,941	69,181	556,028	8,708	149,287	572,567
UL80	pUL80	19,479.66	9	nd	57,699	267,196	nd	160,901	354,602
**Tegument proteins**									
**HCMV ORF**	**Synonym**	**Max score ^a^**	**Reported peptides ^b^**	**RV-HB15**	**RV-HB15**	**RV-HB15**	**RV-BADwt**	**RV-BADwt**	**RV-BADwt**
				**2 hdpi**	**2 dpi**	**4 dpi**	**2 hpi**	**2 dpi**	**4 dpi**
UL25	pUL25	11,575.85	14	5,881	75,440	316,023	15,207	123,334	425,577
UL26	pUL26	2,947.21	2	6,186	17,749	39,969	nd	11,563	39,897
UL32	pp150	1,592.09	10	10,230	10,049	84,106	nd	12,225	76,558
UL35	pUL35	1,480.43	3	nd	12,827	26,106	nd	18,355	28,489
UL45	RR1	765.52	2	nd	7,799	38,594	nd	9,887	21,194
UL47	HMWP-BP	1,950.86	8	11,842	9,849	48,624	5,974	13,690	29,478
UL48	HMW-P	934.58	6	nd	7,967	33,958	3,938	12,393	30,863
UL82	pp71	3,682.85	9	8,167	21,851	78,681	10,465	46,063	81,361
UL83	pp65	73,413.98	27	33,740	227,127	3,026,292	100,786	309,371	3,521,603
UL103	pUL103	2114.97	2	nd	9,058	38,143	nd	16,642	34,996
US22	pUS22	3681.41	15	6,956	80,747	120,418	4,665	99,484	116,925
**Envelope proteins/glycoproteins**									
**HCMV ORF**	**Synonym**	**Max score ^a^**	**Reported peptides ^b^**	**RV-HB15**	**RV-HB15**	**RV-HB15**	**RV-BADwt**	**RV-BADwt**	**RV-BADwt**
				**2 hpi**	**2 dpi**	**4 dpi**	**2 hpi**	**2 dpi**	**4 dpi**
UL75	gH	848.31	4	nd	5,856	57,842	nd	18,448	64,162
UL100	gM	2,800.15	2	nd	5,570	80,032	1,528	61,189	158,290
UL115	gL	3,596.83	2	nd	8,146	19,951	nd	15,905	32,299
**Other virion proteins**									
**HCMV ORF**	**Protein**	**Max score ^a^**	**Reported peptides ^b^**	**RV-HB15**	**RV-HB15**	**RV-HB15**	**RV-BADwt**	**RV-BADwt**	**RV-BADwt**
				**0 dpi**	**2 dpi**	**4 dpi**	**0 dpi**	**2 dpi**	**4 dpi**
IRS1	pIRS1	13,209.55	19	4,284	150,080	138,219	5,178	189,945	114,985
TRS1	pTRS1	10,401.20	18	8,602	97,473	87,607	12,635	133,632	45,993
UL34	pUL34	2,813.53	5	nd	35,364	142,758	9,861	56,510	117,824
UL44	pUL44	70,382.59	20	7,621	379,249	1,477,506	5,522	631,538	1,200,976
UL50	pUL50	1,314.75	2	nd	6,143	40,091	3,617	31,638	45,208
UL52	pUL52	1,623.85	11	2,043	29,069	73,220	3,252	58,778	77,154
UL56	pUL56	1,143.55	2	nd	14,351	29,524	nd	16,585	31,192
UL57	pUL57	2,937.02	23	11,186	36,083	84,431	13,807	40,880	69,838
UL69	pUL69	878.59	5	nd	20,258	36,525	nd	24,015	25,949
UL84	pUL84	8,406.89	13	4,630	23,259	108,290	6,403	59,514	137,353
UL89	pUL89	1,075.02	2	nd	2,815	21,271	nd	nd	40,538
UL97	pUL97	1,740.41	4	nd	10,305	33,074	nd	14,152	35,229
UL98	pUL98	2,791.21	9	7,904	18,894	53,714	1,835	31,351	89,966
UL112/113	pUL112/113	4,259.03	9	39,266	88,103	163,205	39,268	68,815	160,294
UL122	IE2	38,858.88	9	4,333	68,426	309,712	4,071	101,350	431,908
UL123	IE1	2,927.93	7	1,829	33,285	2,040	3,456	25,160	7404

^a^ maximum Protein Lynx Global Server (PLGS) identification score; Tandem MS Search Algorithm [35]; ^b^ number of different peptides detected for each protein in all samples; ^c^ TOP3 quantification values correlated with the protein amounts; nd, not detected.

### 2.3. Conserved Stoichiometry of Viral Proteins in Virions of AD169-Derived Viruses

**Figure 4:**
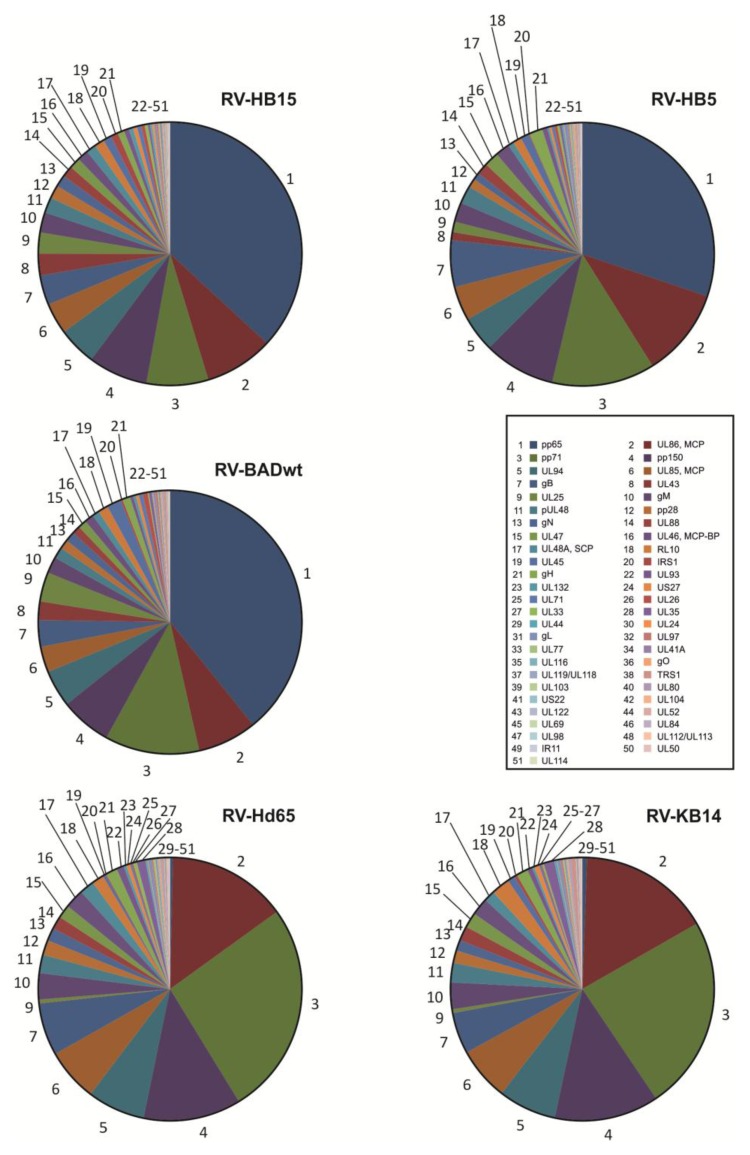
Proteomic analysis of HCMV proteins from purified virions. Virions were purified by glycerol-tartrate-gradient-centrifugation from supernatants of 6–7 day infected foreskin fibroblasts. The pp65pos strains (RV-HB5, RV-HB15, and RV-BADwt) and the pp65neg strains (RV-Hd65 and RV-KB14) were used for analysis. Purified virions were analyzed using nanoUPLC mass spectrometry. The stoichiometry of individual proteins, normalized to exogenously added enolase is shown in the pie charts. Proteins were sorted in decreasing abundance according to the values obtained for RV-HB15 virions and numbered accordingly, as indicated in the figure caption.

Viral protein levels in HFF were comparable between the two AD169-derived viruses. We next asked the question, if this conservation was also reflected in the proteomes of virions from the different viruses. For this, also two AD169 variants were used that did not express the most abundant tegument protein pp65, in addition to three pp65positive (pp65-pos) viruses. Proteomic analyses showed a striking level of similarity in the protein pattern of the three pp65-pos viruses (RV-HB5; RV-HB15; RV-BADwt) and the two pp65-negative (pp65-neg) viruses (RV-Hd65; RV-KB14), respectively (Figure 4). As expected, the most prominent constituent of the pp65pos viruses was pp65, followed by the major capsid protein, pp71, pp150 and pUL94. These results were comparable to what has been published before for the proteome of AD169 virions [25]. There was some variation in the relative molarity of pp65, which is a non-essential tegument protein [18]. The number of pp65 molecules that are included in the tegument may thus vary. Accordingly, the relative copy numbers of some of the tegument proteins, known to interact with pp65 [36,37,38] showed subtle alterations which may be related to variations in pp65 content. There were also some differences in the ranking of abundance of some other virion proteins, compared to previously published data [25]. We do not know the reasons for this at this point. Different infection and particle purification strategies, e.g., with regard to the time point of collection of infectious supernatant or with regard to ultracentrifugation may account for these variations. In addition, different mass spectrometry protocols were used. In our study, exogenously added enolase was employed as an internal standard, providing a high level of confidence with respect to relative quantification. However, more detailed analyses of the virion composition, including different strains, will have to be performed to more accurately display relative abundances of individual proteins in HCMV virions.

The compositions of the two pp65neg viruses and the three pp65pos viruses were virtually identical, except for the lack of pp65 in the former (Figure 4). Note that RV-Hd65 is a derivative of AD169‑BAC [29], the BAC, used for reconstitution of RV-HB15 (AD169-RV), and RV-KB14 is a derivative of pAD/cre [30], the BAC used for reconstitution of RV-BADwt. The slight differences seen in the protein pattern of the two parental viruses were not detectable in the pp65neg variants. 

Taken together these results showed that the viral protein composition of extracellular virions of AD169 descendants is subject to only subtle variations. This argues in favor of a conserved process of viral protein packaging within a given strain of HCMV. Remarkably, however, the pattern did not change dramatically by removing pp65 which, in our analyses, comprised up to one third of the total mass of viral proteins in the particle. Despite the absence of pp65, tegument assembly appeared to follow along a controlled process that resulted in protein patterns that were comparable between pp65pos and pp65neg viruses. It is likely that the network of tegument protein interactions, as reported by others [37,39] will be instrumental to this process; yet this hypothesis still awaits confirmation. 

## 3. Experimental Section

For proteomic analyses of infected cells, HFF were infected at a multiplicity of infection (MOI) of 1 in each case. For this, culture supernatants from 6–7 day infected HFF had been collected and stored at −80 °C. One sample was thawed and analyzed for infectivity, using IE1 (UL123) specific antibody staining. An MOI, based on serial dilution of the supernatants was calculated. For proteomic analyses of virions, cells were infected in a way that all cells showed a cytopathic effect at day one of infection.

Mass spectrometry was performed as described elsewhere [32]. Briefly, sample preparation and protein digestion of virions were performed as described in [32]. Nanoscale LC separation of tryptic peptides was performed with a nanoAcquity system (Waters Corporation, Manchester, UK) equipped with a BEH C18 1.7 μm, 75 μm × 150 mm analytical reversed-phase column (Waters Corporation) in direct injection mode as described before [40]. Mobile phases, gradients and flow rates were chosen as described in [32] and 0.2 μL of sample (50 ng of total protein) was injected per technical replicate. Running conditions were as described in [32].

Mass spectrometric analysis of tryptic peptides from infected cells and virions was performed in quintuplicate using a QTOF-Premier mass spectrometer (Waters Corporation) with a typical resolution of at least 10,000 FWHM (full width half maximum) as described before [32]. All analyses were performed in positive mode ESI using instrument settings and nanoLockspray calibration as described before [32].

Continuum LC-MS data were processed and searched using ProteinLynx GlobalSERVER version 2.5.2 (Waters Corporation) [41]. Protein identifications were obtained by searching a custom compiled database containing sequences of human and HCMV proteins from the Uniprot database. Sequence information of enolase 1 (*S. cerevisiae*) and bovine trypsin were added to the databases to normalize the data sets or to conduct absolute quantification as described before [42]. Database search was performed allowing a maximal mass deviation of 15 ppm for precursor ions and 30 ppm for fragment ions with one missed cleavage allowed and fixed carbamidomethyl-cysteine and variable methionine oxidation set as the modifications. For valid protein identification, the following criteria had to be met: at least two peptides detected with together at least seven fragments. The false positive rate for protein identification was set to 1% based on search of a triple randomized database. Guideline identification criteria were applied for all searches. 

For the absolute quantification of proteins, we employed a well established label-free quantitative proteomics workflow that we have previously used both for the quantification of nanoparticle protein coronas [40] and also higher complexity samples such as myelin [42]. Label-free quantification using the TOP3-approach allows both the relative and absolute quantification of proteins. TOP3-Intensity was calculated as the average intensity of three best ionizing peptides and is proportional to the molar amount of the respective protein in the sample [43]. Values were normalized across technical replicates and samples using ISOQuant [44]. For the proteomic analysis of HFF, cells were infected at an MOI of 1 for 2 h, 2 d, or 4 d, respectively. After that, cells were collected by removing the culture supernatant and subsequently adding 5 mL of 50 mM EDTA solution in 1× PBS. They were detached from the support after an incubation time of 5–15 minutes at 37 °C and were subsequently centrifuged at 470 g for five minutes. The pellet was resuspended in 10 mL of PBS and the cells were centrifuged again as described above. This was repeated once. The final pellet was resuspended in 2–3 mL PBS and the cells were counted. 0.5 millions of cells each were transferred to 1.8 mL Eppendorf tubes and the cells were again centrifuged at 1,300 g for three minutes. The supernatant was carefully removed and the tubes were stored at −80 °C until further mass spectrometry. The samples were subsequently analyzed by mass spectrometry as detailed above.

Virions were purified from the culture supernatants of HCMV infected human foreskin fibroblasts (HFF). For this, cells were infected in a way that all cells showed a typical cytopathic effect at day 1 after infection. Culture supernatants were collected at 6–7 days of infection and were purified by gradient ultracentrifugation as originally described by Irmiere and Gibson [15]. For this, the culture supernatants were collected and centrifuged for 10 min at 1,900 g to remove cells and debris. After that, the supernatant was collected and centrifuged at 95,000 × g (70 min, 10 °C) in a SW32Ti rotor in a Beckman Optima L-90K ultracentrifuge. Pellets were resuspended with 2 mL 1× PBS. Na-tartrate gradients were prepared directly before use. For this, 4 mL of 35% Na-tartrate solution in 0.04 M Na‑phosphate buffer, pH 7.4 were applied in one, and 5 mL of 15% Na-tartrate/30% glycerol solution in 0.04 M Na-phosphate buffer, pH 7.4 were applied in the second column of a gradient mixer. The gradients were prepared by slowly dropping the solutions into Beckman Ultraclear^TM^ centrifuge tubes (14 × 89 mm), positioned at an angle of 45°. 1 mL of the viral particles was then carefully layered on top of the gradient. Ultracentrifugation was performed in slow deceleration mode in a Beckman SW41 swing out rotor for 60 min at 90,000 g and 10 °C. Particles were collected from the gradient, illuminated by light scattering. For this, the centrifuge tube was penetrated with a hollow needle below the band and samples were carefully drawn from the tube with a syringe. The particles were then washed once with 1× PBS and centrifuged in a SW41 swing out rotor for 90 min at 100,000 g and 10 °C. Following that centrifugation step, the pellets were resuspended in 120–150 µL 1× PBS. The protein concentration of the purified virions was determined with the Pierce BCA Protein Assay Kit (Thermo Scientific, Bonn, Germany). Twenty µg aliquots of the virions were then pelleted by ultracentrifugation for 60 min, 100,000 g at 10 °C and stored at −80 °C for analysis by mass spectrometry. 

## 4. Conclusions

We are only at the advent in our understanding of the complexity of HCMV particle morphogenesis. Mass spectrometry has provided a technical leap in our attempts to understand the molecular events that direct the formation and release of infectious progeny. The results indicate that there are only limited variations in both the intracellular and the virion proteome with regard to viral proteins in derivatives of one particular laboratory strain, AD169. This argues in favor of a highly ordered process of viral protein expression and interaction to ultimately result in packaging and virion release. In line with this, also the proteome of cellular proteins appeared to be conserved following infection with different viruses. However, the data also immediately foster the request for analogous proteomic analyses of other HCMV strains, clinical isolates being of particular interest in this respect.

## Supplementary Files

Supplementary File 1 Supplementary Information (XLSX, 395 KB)
